# Gene Dysregulation in the Adult Rat Paraventricular Nucleus and Amygdala by Prenatal Exposure to Dexamethasone

**DOI:** 10.3390/life12071077

**Published:** 2022-07-19

**Authors:** Tyler R. Rivet, Christine Lalonde, T. C. Tai

**Affiliations:** 1 School of Natural Sciences, Laurentian University, Sudbury, ON P3E 2C6, Canada; trivet@laurentian.ca; 2 Department of Psychology, Nipissing University, North Bay, ON P1B 8L7, Canada; chrisl@nipissingu.ca; 3 Biomolecular Sciences, Laurentian University, Sudbury, ON P3E 2C6, Canada; 4 Division of Medical Sciences, NOSM University, Sudbury, ON P3E 2C6, Canada

**Keywords:** glucocorticoids, dexamethasone, fetal programming, amygdala, paraventricular nucleus, HPA axis, behavior

## Abstract

Fetal programming is the concept that maternal stressors during critical periods of fetal development can alter offspring phenotypes postnatally. Excess glucocorticoids can interact with the fetus to effect genetic and epigenetic changes implicated in adverse developmental outcomes. The present study investigates how chronic exposure to the synthetic glucocorticoid dexamethasone during late gestation alters the expression of genes related to behavior in brain areas relevant to the regulation and function of the hypothalamic–pituitary–adrenal axis. Pregnant Wistar Kyoto rats received subcutaneous injections of dexamethasone (100 μg/kg) daily from gestational day 15–21 or vehicle only as sham controls. The amygdala and paraventricular nucleus (PVN) were micro-punched to extract mRNA for reverse transcription and quantitative polymerase chain reaction for the analysis of the expression of specific genes. In the PVN, the expression of the glucocorticoid receptor *NR3C1* was downregulated in female rats in response to programming. The expression of *CACNA1C* encoding the Ca_v_1.2 pore subunit of L-type voltage-gated calcium channels was downregulated in male and female rats prenatally exposed to dexamethasone. Collectively, the results suggest that prenatal exposure to elevated levels of glucocorticoids plays a role in the dysregulation of the hypothalamic–pituitary–adrenal axis and potentially learning and memory by altering the expression of specific genes within the amygdala and PVN.

## 1. Introduction

The thrifty phenotype hypothesis put forth by Barker and Hales in 1992 attempted to explain the development of impaired glucose tolerance and later metabolic syndrome with respect to impaired growth in fetal and infantile stages of development [1,2,3]. Their research was among the first to describe the consequences of fetal programming. In fetal programming, stressful conditions in the external environment producing maternal distress are effectors of phenotypic alterations to progeny postnatally [3,4,5]. These alterations are not necessarily adaptive and tend to contribute to the development of disease in later life.

Other sources of fetal programming include fetal glucocorticoid (GC) exposure altering hypothalamic–pituitary–adrenal (HPA) axis activity [6]. These GCs may be endogenous or synthetic, as with dexamethasone (Dex) administered to mothers at risk for preterm labor.

The HPA axis is the physiological system responsible for the secretion of GCs such as cortisol in humans. Adrenocorticotropic hormone (ACTH) released from the anterior pituitary gland stimulates cellular activity, promoting the synthesis of cortisol from cholesterol in the adrenal cortex [7]. During an acute response to an immediate stressor, the amygdala processes the initial threatening stimuli, signaling the hypothalamus, which subsequently activates the sympathetic nervous system, keeping the organism in an alert and energized state for a reaction to the initial stressor [8]. If it is necessary to maintain this state over a longer period, the paraventricular nucleus (PVN) of the hypothalamus initiates HPA axis signaling, causing the release of ACTH from the anterior pituitary and downstream cortisol production [9,10].

During gestation, the fetus is not typically exposed to cortisol until the end of the pregnancy, when the fetal adrenal glands begin to produce it [11]. Due to the action of a placental enzymatic barrier, cortisol is unidirectionally converted to more inert cortisone by the enzyme 11β-hydroxysteroid dehydrogenase 2 in the placenta [12]. Under normal physiological conditions, this placental barrier is effective in shielding the fetus from the deleterious effects of prenatal GC exposure [13]. However, synthetic GCs and heightened chronic maternal stress have been shown to bypass or overwhelm this barrier [6,14,15,16,17,18]. By bypassing the barrier, GCs may interact with fetal tissues relatively unhindered. Fetal exposure to endogenous or synthetic GCs in this capacity has been implicated in the programming of hypertension [17,19,20], mental illness [15,16,21], and altered brain morphology [22,23].

The brain rapidly develops and matures during gestation, leaving it particularly susceptible to damage by environmental factors [24,25]. In recent years, particular focus has been on fetal programming as a mediator of the development of adverse mental health outcomes. For example, elevated maternal cortisol, administered synthetic GCs, and maternal depression have been shown to elicit depressive symptoms in offspring [15,16,26]. Molecules in the plasma are typically unable to interact with the brain owing to the presence of a blood–brain barrier, functional as early as 8 weeks in gestation [27]. However, endogenous GCs are able to diffuse into the central nervous system, with efflux transporter p-glycoprotein playing only a minor role, while synthetic GCs are more hindered [28,29,30]. Conversely, there are certain organs within the brain that circumvent the blood–brain barrier, aptly named the circumventricular organs. Areas such as the subfornical organ are in direct contact with other regions protected by this barrier and provide an alternative pathway for hormonal communication [31,32]. There is also evidence to suggest that behavioral tendencies may be programmed as well. Rats exposed to Dex prenatally have shown increased immobility on the Porsolt swim test (PST), a measure of learned helplessness and despondency, among other behaviors likened to a depressive presentation [18,33,34]. Similarly, in humans, elevated maternal GCs have corresponded to the presence of affective issues and internalizing behaviors [16,23,35]. Taken together, evidence tends to suggest that prenatal Dex exposure has diverse consequences with respect to offspring brain morphology and behavior. The programming of limbic and HPA axis-related structures in these cases is associated with affective issues such as depressive symptoms.

GR expression is high in the PVN, the nucleus of the hypothalamus that initiates the HPA axis [36]. Di et al. (2003) demonstrated that Dex ligands elicit the rapid inhibition of the excitatory glutamate release into the PVN of parvocellular neurosecretory neurons that secrete corticotropin-releasing hormone (CRH) [37]. The experiment provided evidence of a rapid feedback inhibition mechanism by GCs on PVN CRH secretion. CRH secretion causes the release of ACTH from the anterior pituitary gland, in turn allowing for cortisol secretion by the adrenal cortex [38].

**Figure 1:**
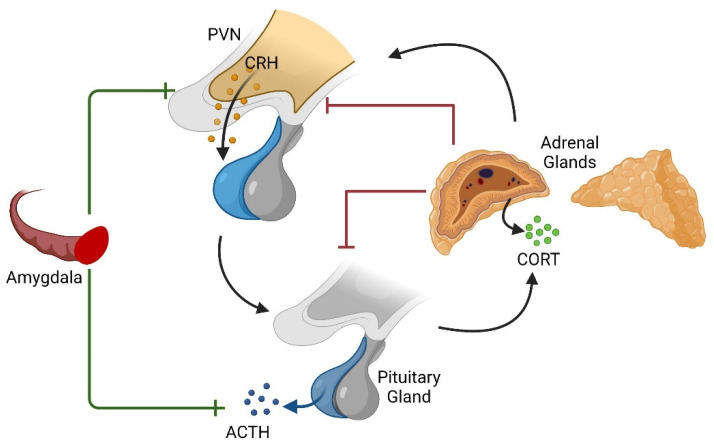
Diagrammatic representation of the HPA axis and modulation by the amygdala. While endogenous GCs provide a negative feedback mechanism after their production in the adrenal gland and the occupation of GC receptors, the amygdala is known to stimulate the axis upstream of the adrenal glands. Created with BioRender.com accessed on 23 June 2022.

Another brain region of particular interest in relation to anxiety- and fear-related behaviors is the amygdala, a structure composed of several distinct nuclei in the medial temporal lobes. Functions such as signaling emotional and physiological response to fearful stimuli like freezing and instrumental behaviors like fleeing involve the central and basal nuclei, respectively [39]. Increases or decreases in transmission between these two nuclei have also been demonstrated to augment or inhibit anxiety-related behaviors, respectively [40,41]. Notably, the amygdala is also known to increase the stress-related secretion of GCs by the HPA axis, contrary to the hippocampus’s role in inhibiting stress-induced HPA activation and reducing GC secretion (Figure 1) [42,43,44].

We have previously shown that Dex administration to pregnant Wistar–Kyoto rats as a proxy for late gestational stress can elicit changes in the gene expression in the offspring prefrontal cortex (PFC), as well as in behavior [18]. Behavior observed on the Porsolt swim test included the greater immobility of the Dex-programmed offspring relative to controls on the first day of testing, while on the second day, female rats in both treatment and control conditions displayed increased immobility relative to males. The elevated plus maze (EPM) is a well-validated test whereby anxiety-like behaviors, or the lack of these, can be measured based on the typical rodent’s proclivity for dark, enclosed spaces and locations away from observable heights [45]. Dex-programmed animals entered the open arms with 4 paws more often than the sham-treated offspring, while entering the enclosed arms with 2 paws less often, reflecting a reversal of expected normal thigmotaxic behaviors [18,46]). In this study, we measured changes in gene expression in the PVN and amygdala of the same Dex-programmed animals from the original study.

Given what is known about GCs and their role in the fetal programming of the brain and behavior, the question arises as to how GC programming may contribute to HPA axis dysregulation and the production of maladaptive behaviors, [38,47,48,49]. Two brain areas, the PVN and amygdala, were selected for their richness in GRs, relevance to regulating the HPA axis, and roles in behavior. Considering the evidence at hand, we hypothesize that exposure to synthetic GCs prenatally programs the dysregulation of gene expression in the amygdala and PVN, with potential implications for behavioral outcomes.

## 2. Materials and Methods

Experimental protocols involving the use of animals were approved by the Animal Care Committee of Laurentian University (AUP: 6013917) and followed guidelines established by the Canadian Council on Animal Care. Eight-week-old Wistar–Kyoto rats purchased from Charles River Laboratories (Saint-Constant, Québec, Canada) were housed in groups of two or three in Innocage^®^ IVC disposable cages (Innovive Inc., San Diego, CA, USA). Cob bedding (Harklan, Madison, WI, USA) as well as Bio-serv enrichment tubes (Frenchtown, NJ, USA) were present in the cages. Cages were kept in a HEPA-filtered Innorack^®^ rat airflow system (Innovive Inc., San Diego, CA, USA). Both food (Teklad 22/5 rodent diet, Harklan, KY, USA) and water were made available to the animals *ad libitum*. A 12-h day–night cycle was instituted, with the former beginning at 6:00 a.m. Ambient room temperature was maintained at 25 °C with 53% relative humidity.

Male rats were introduced singly per three females over a period spanning five days, after which female rats were checked for the presence of a vaginal plug to confirm the occurrence of mating. Pregnant females were housed alone and weighed daily for the duration of their gestational span. Upon gestational day fifteen, and every day thereafter, pregnant rats received one of two treatments. Four dams were selected for the exposure group and were subjected to daily subcutaneous injections of Dex (Sigma-Aldrich, Milwaukee, WI, USA; 0.9% sodium chloride, 4% ethanol, and 100 μg per kg body mass Dex), while another dam received vehicle only to produce sham controls. Injections were given during the light cycle, as previously described [17,18,20].

Pups were raised normally and weaned at three weeks of age. Eight pups were selected per sex from the four Dex-exposed dams, while eight pups per sex were selected from the sham exposure mother. Upon reaching nineteen weeks of age, young adult rats were euthanized by intraperitoneal injection of 75 mg ketamine (100 mg/mL, Ketalean, CDMV Inc., Brampton, ON, Canada) and 5 mg xylazine (100 mg/mL, Sigma, Milwaukee, WI, USA) per kilogram of body mass. Tissues of interest were collected immediately and subsequently frozen using dry ice. Whole brains were collected and stored at −80 °C for future genetic and morphological analyses. Coronal sections of rat brains with widths of 5–20 μm were made using a CM3050 S cryostat (Leica Biosystems, Deer Park, IL, USA) maintained at −20 °C. Using the rat brain atlas of Paxinos & Franklin (1997), areas of interest were identified, and anterior–posterior measurements from bregma were used to delineate their locations in the sections [50]. Punctures in the sections were made using a sterile pipette tip to retrieve relevant tissues within the Cryostat.

The tissues retrieved from the right amygdala and the paraventricular nucleus of each rat were placed in TRI reagent (Sigma-Aldrich, Oakville, ON, Canada; 1 mL/50 mg of tissue) and subjected to two 2-min cycles at 30 hertz in a TissueLyser (Qiagen, Hilden, Germany) for the mechanical homogenization of the sample. mRNA was then extracted from the samples using a TRIzol extraction method, as previously described [18].

Using Primer3 and BLAST, primers for use in real-time quantitative polymerase chain reaction (PCR) were designed for the experiment (Table A1). As per Livak and Schmittgen (2001), primers were validated using serial dilutions of cDNA and amplified in PCR [51]. The specificity of primers was evaluated using melt curves post amplification. Genes of interest were selected based on a whole-genome microarray analysis of the brain [52] and included those relevant to the metabotropic glutamate receptor pathway, calcium signaling, neural differentiation and growth, and glucocorticoid receptors. Two reference genes, *CycA* and *Ywhaz*, were selected based on Bonefeld et al. [53].

Relative gene expression between treatments (n = 8 per sex per treatment) was analyzed using the QuantStudio5 Real-Time PCR System (Applied Biosystems, Foster City, CA, USA). Amounts of 15 μL reaction volume per sample in 96-well plates contained 0.4 ng/μL cDNA, DEPC-treated water, 1.2 μM/μL forward and reverse primers for each respective gene, and SYBR green master-mix (SensiFAST SYBR Lo-ROX, Bioline, FroggaBio, Toronto, ON, Canada). Changes in relative gene expression between treatments with respect to reference genes, with the sham male group as a calibrator, were measured using the ΔΔC_T_ method, followed by melt curve analysis [51].

Statistical analyses for genetic comparisons between groups were conducted using Jamovi v1.6.23 and R version 4.1.3 [54,55]. The parametric assumptions of normality and homoscedasticity in analysis of variance (ANOVA) were tested using the Shapiro–Wilk and Levene’s tests, respectively. Where the assumption of homoscedasticity was rejected between treatments, Welch’s test was conducted. Where both assumptions were met, two-factor ANOVAs with interaction were conducted. Where primary effects of treatment were determined in ANOVA, Dunnett’s post hoc test was employed to assess differences in gene expression between rats born to Dex-exposed mothers and their respective controls. Where primary effects were a result of sex rather than treatment, Tukey’s post hoc test was employed to assess differences in gene expression between male and female control rats as well as male and female rats born to Dex-exposed mothers.

## 3. Results

### 3.1. Paraventricular Nucleus

**Figure 2:**
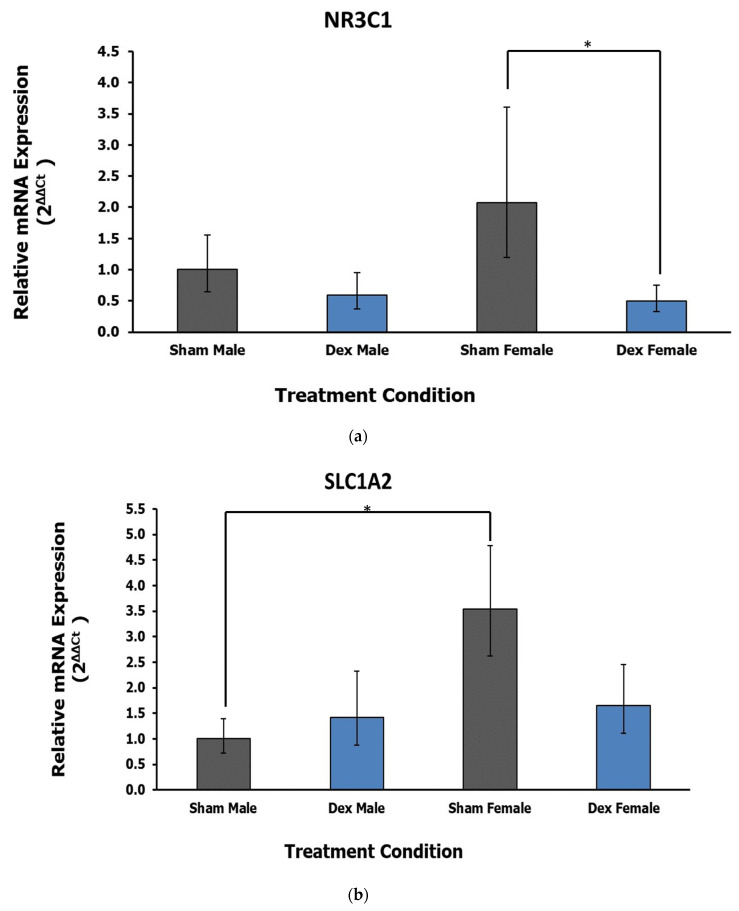
(**a**) Relative fold change in the PVN gene expression of glucocorticoid receptor NR3C1. Dex condition female rats show a decrease in the expression of this gene relative to control. Error bars represent a 95% confidence interval. * = *p* < 0.05. (**b**) Relative fold change in the PVN gene expression of SLC1A2. Female rats overall display higher expression of this gene compared with males independent of treatment. A potential interaction trend is noted but is not statistically significant. * = *p* < 0.05.

Of the fifteen genes selected for analysis, two of them displayed dysregulation as an effect of treatment in the PVN (Table 1). A significant effect of treatment was demonstrated by ANOVA for the expression of the Nuclear Receptor Subfamily 3 Group C Member 1 (*NR3C1*; F_1,17_ = 6.81, *p* = 0.018, η^2^ = 0.263). No significant effect of animal sex (F = 0.522, *p* = 0.480, η^2^ = 0.020) or interaction was noted (F = 1.509, *p* = 0.236, η^2^ = 0.058) Post hoc Dunnett’s test reveals a significant decrease in *NR3C1* expression in Dex condition female rats relative to respective sham control (*p* = 0.031; Figure 2a). The gene encoding Glutamate Ionotropic Receptor AMPA Type Subunit 2 (*GRIA2*) shows a significant effect of treatment in ANOVA (F_1,19_ = 5.40, *p* = 0.031, η^2^ = 0.212). However, Dunnett’s test indicates that differences in expression between Dex condition animals showed a trend but were not statistically significant at *p* < 0.05, compared with sham controls. Solute Carrier Family 1 Member 2 (*SLC1A2*) displayed a significant effect of sex (F_1,19_ = 5.24, *p* = 0.034, η^2^ = 0.187) rather than treatment (F = 0.446, *p* = 0.512, η^2^ = 0.016). In this case, a sex–treatment interaction trend is noted but is not significant, at F = 3.282, *p* = 0.086 and η^2^ = 0.117. Sham females appear to have higher baseline expression of *SLC1A2* that is attenuated in the Dex exposure condition, whereas lower baseline expressing males appear to display slight upregulation in the Dex exposure condition. Post hoc Tukey’s test indicates that sham control females have higher expression of this gene compared with male rats (*p* = 0.036; Figure 2b). Notably, this difference between sham female and male rats is attenuated in both of the Dex exposure conditions.

### 3.2. Amygdala

**Figure 3:**
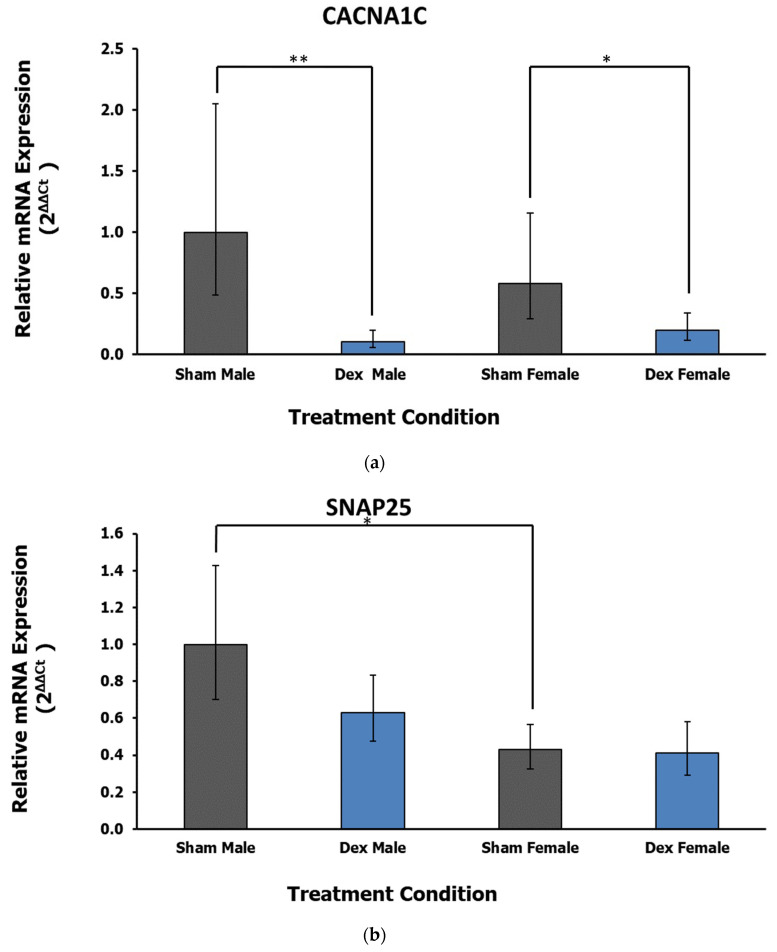
(**a**) Relative fold change in the amygdala gene expression of CACNA1C. A main effect of treatment is the downregulation of this gene in both Dex condition males and females relative to respective controls. Error bars represent a 95% confidence interval. * = *p* < 0.05, ** = *p* < 0.01. (**b**) Relative fold change in the amygdala gene expression of *SNAP25*. Relative to males, female rats display lower levels of gene expression. Error bars represent a 95% confidence interval. * = *p* < 0.05.

In the amygdala, the expression of one gene related to calcium signaling is dysregulated in relation to treatment, while another sex difference was observed in a neural transmission gene. Calcium Voltage-Gated Channel Subunit Alpha 1 C (*CACNA1C*) displays a significant effect of treatment (F_1,26_ = 17.87, *p* < 0.001 η^2^ = 0.388) with no significant effect of sex (F = 0.01, *p* = 0.917, η^2^ = 0) or interaction (F = 2.17, *p* = 0.152, η^2^ = 0.047). Post hoc Dunnett’s test reveals the downregulation of *CACNA1C* in both Dex condition male (*p* = 0.004) and female (*p* = 0.043) rats relative to respective controls (Figure 3a). Finally, the gene encoding the Synaptosome-Associated Protein, 25 kDa (*SNAP25*) displays a significant effect of sex (F_1,25_ = 10.57, *p* = 0.003, η^2^ = 0.275) rather than treatment (F = 1.67, *p* = 0.208, η^2^ = 0.044) or interaction (F_x,y_ = 1.17, *p* = 0.289, η^2^ = 0.031). Post hoc Tukey’s test reveals a relative decrease in expression in female sham controls relative to male rats (*p* = 0.028, Figure 3b). Notably, the difference in expression between sham control animals is lost in the Dex exposure conditions.

## 4. Discussion

Traditionally known for its role in the HPA axis, recently the PVN is receiving further attention for its roles in modulating the emotional and anxiety networks as well as anxiety- and depression-like behaviors [56]. Another key player in these networks is the amygdala, with specific nuclei having excitatory roles in increasing HPA axis activity [9]. With normal neural development, these areas of the brain are integral to adaptive behaviors in response to stressful stimuli. It is expected that alterations in these areas as a result of fetal programming would alter key features in adaptive behaviors.

Differential expression of genes was observed in the PVN as an effect of treatment. *NR3C1*, encoding the GR, was downregulated in Dex condition female rats specifically. This effect is thought to reflect a decrease in negative feedback to the PVN by GCs that would facilitate and maintain the HPA axis response to stressors [57]. Chronic perinatal exposure to GCs has previously been shown to decrease GR expression in the PVN, raising CRH and endogenous GC production with implications for the development of mood and anxiety disorders [58,59,60]. Another study using similar conditions to those described here reported GR downregulation in the hippocampus of WKY rats that also involved negative feedback of the HPA axis, as suggested by elevated levels of corticosterone in animals born to Dex-treated mothers [61]. Results such as these tend to correspond with increases in circulating endogenous GCs, indicating overactivity of the HPA axis [62]. For example, Dex-programmed vervet monkeys demonstrate elevated blood cortisol levels following mild stress compared with controls [63]. A similar presentation is observed in prenatally stressed rhesus monkeys by Schneider et al. (2002), accompanied by reduced locomotion and exploratory activity [64]. A more robust activation of the HPA axis is often reported in female animals compared with males, with estrogens implicated as modulating HPA axis activity by decreasing negative inhibition of the axis in the central nervous system, including the PVN [65,66]. It is thought that females may be more sensitive to the effects of gestational exposure to stress hormones as a result [67]. The results of this study may indicate that fetal programming by GCs causes dysregulation of the HPA axis in female rats selectively, with estrogens contributing to observed differences in sex. While sex differences associated with Dex programming were not apparent in behavioral testing in the previous study, behaviors described as adaptive in response to stress were observed in general for animals born to Dex-exposed mothers in the PST and EPM [18]. Alterations in HPA axis activity and regulation may support adaptive stress-related behavior in response to adverse conditions, such as the PST, though the current results do not support a mechanism explaining altered behavior in programmed male rats.

The expression of *GRIA2*, encoding a subunit of the AMPA-type glutamate receptor (AMPAR), was upregulated in Dex-condition offspring non-specifically within the PVN. AMPARs are glutamate-gated heterotetrameric ion channels mediating most of the fast excitatory synaptic transmission in the brain [68,69]. AMPARs have been well studied in the processes of memory and learning, though their role elsewhere is poorly understood [70]. Hypothalamic AMPARs facilitate autonomic responses to stressors, such as increases in blood pressure, where the enrichment of PVN neurons with AMPARs lacking GRIA2 increases excitability related to hypertension [71,72]. Reductions in AMPAR transmission have been implicated in anxiety and stress, while disruption in the trafficking of the GRIA2 subunit has been associated with predisposition to stress [71,73,74,75]. Finally, positive allosteric modulators of AMPARS produce antidepressant effects and have improved behavioral and glutamate transmission deficits in rats stressed perinatally [74,76,77]. Targeting hypothalamic AMPARs for novel therapies in diverse disorders has been proposed, though the specific roles maintained by the GRIA2 subunit in these processes are currently understudied [70].

The downregulation of *CACNA1C*, encoding the Ca_v_1.2 pore subunit of L-type voltage-gated calcium channels (L-VGCCs), occurred as an effect of treatment in both Dex-condition males and females in the amygdala. This finding contrasts a previous in vitro experiment where the direct application of GCs to basolateral amygdala neurons increased the expression of the Ca_v_1.2 subunit [78]. The mechanism linking Dex exposure in gestation and the reduction of *CACNA1C* in the entire amygdala likely differs considering the current results.

The role of L-VGCCs has also been studied in fear conditioning and memory in the lateral amygdala. During membrane depolarization, influxes of calcium through the NMDA-type and L-VGCCs are thought to underlie the processes of long- and short-term fear memory formation [79,80]. The inhibition of L-VGCCs, however, impairs the formation of long-term fear memories only [80,81,82]. Similarly, the activity of L-VGCCs is required in the basolateral amygdala for the occurrence of long-term fear extinction [83]. *CACNA1C* region-specific knockouts and heterozygous mice have displayed a variety of other impairments in different facets of memory, including spatial memory [84]. Deficits in the expression of the Ca_v_1.2 subunit in the amygdala may reflect similar memory-deficit phenotypes in rats born to Dex-exposed mothers. Behavioral testing paradigms in the original study were not designed for the detection of fear-related learning and memory, though results obtained in this study indicate such inquiry may be warranted.

Two effects in gene expression by sex were observed in this study that were abolished by treatment. In the PVN, the expression of *SLC1A2* was higher in female rats compared with males in the sham control conditions. The encoded membrane-bound protein removes glutamate from the synaptic cleft in the central nervous system, playing an important role in staving off neuronal excitotoxicity [85]. Sex differences in *SLC1A2* mRNA expression have not yet been reported to the best of our knowledge. The other effect of sex on gene expression was found within the amygdala, where *SNAP25*, an integral component of neuronal vesicle exocytosis and docking, was expressed at higher levels in male rats compared with female rats [86]. Both splice variants of the SNAP25 protein, SNAP25a and SNAP25b, are mostly confined to neurons of the central nervous system [87]. The upregulation of the *SNAP25* gene was previously reported in the PFC of male rats born to Dex-exposed mothers [18]. Sexually dimorphic expression of SNAP25 has been proposed as one underlying feature of sex differences in brain function [88]. For example, the incidence of attention deficit hyperactivity disorder is higher in males, which may reflect sexually dimorphic differences in frontal lobe functioning. As for the role of SNAP25 in the amygdala, further study is required to determine what function sex differences in gene expression may serve. The loss of significant differences in the expression of *SLC1A2* and *SNAP25* in the Dex exposure conditions is also of interest and warrants further exploration.

While behavioral data is useful for corroborating results, the testing paradigms from our earlier study were not designed for the thorough evaluation of processes like learning, fear conditioning and extinction, and responses to stressful situations [18]. A wide array of processes possibly influenced by GC fetal programming in different areas of the brain should be considered in designing future experiments. Evaluating differences in fetal programming modified by sex and sex hormones, especially as they pertain to the HPA axis, also represents a logical target for study given the sex differences in gene dysregulation reported here.

## 5. Conclusions

The present study demonstrates that two brain areas that have not received as much attention in fetal programming studies, the PVN and amygdala, are relevant targets for studies on stress programming. Chronic prenatal exposure to GCs in WKY rats alters gene transcription in these areas of the brain that are relevant to regulating the HPA axis, behavior, emotion, and memory, among other features related to the development of mental illness. Further study of how these areas of the brain are impacted by stress programming later in life may provide further evidence to explain their roles in the development of maladaptive behavior and psychiatric illness.

## Figures and Tables

**Table 1:** Relative fold change gene expression in offspring born to dex-exposed mothers by sex relative to sham control. Where values are shaded in red, a significant primary effect of treatment was identified. Where values are shaded in blue, a significant primary effect of sex was identified.

Gene	Sex	Fold Change Amygdala	Fold Change PVN
		(2^ΔΔCT^ ± SE)	(2^ΔΔCT^ ± SE)
Glucocorticoid Receptor			
NR3C1	Male	1.38 (1.02, 1.86)	0.60 (0.37, 0.95)
	Female	1.21 (0.81. 1.79)	0.24 (0.14, 0.40)
Glutamate Signalling			
GRIA2	Male	1.20 (0.91, 1.58)	2.43 (1.49, 3.94)
	Female	0.88 (0.76, 1.02)	2.00 (1.28, 3.12)
SLC1A2	Male	0.57 (0.41, 0.80)	1.46 (0.87, 2.36)
	Female	1.05 (0.87, 1.28)	0.47 (0.33, 0.68)
Calcium Signalling			
Ryr2	Male	0.65 (0.35, 1.22)	NA
	Female	0.92 (0.72, 1.16)	
PLCH2	Male	0.97 (0.81, 1.17)	1.72 (1.07, 2.77)
	Female	1.45 (1.23, 1.71)	0.98 (0.57, 1.66)
CACNB2	Male	0.27 (0.15, 0.48)	1.85 (1.06, 3.21)
	Female	0.92 (0.59, 1.44)	0.76 (0.50, 1.14)
CACNA1B	Male	1.24 (1.06, 1.46)	0.91 (0.56, 1.51)
	Female	1.22 (0.94, 1.58)	1.19 (0.90, 1.58)
CACNA1C	Male	0.11 (0.07, 0.20)	1.41 (0.87, 2.29)
	Female	0.34 (0.21, 0.55)	1.82 (1.37, 2.24)
CAM2KA	Male	1.14 (0.74, 1.74)	0.89 (0.64, 1.25)
	Female	0.83 (0.68, 1.03)	1.36 (1.16, 1.59)
Neural Transmission			
SNAP25	Male	0.63 (0.47, 0.83)	1.28 (0.95, 1.74)
	Female	0.96 (0.74, 1.24)	0.94 (0.85, 1.04)
Synaptophysin	Male	1.04 (0.85, 1.27)	0.91 (0.65, 1.28)
	Female	1.10 (0.97, 1.24)	1.06 (0.52, 2.17)
Growth and Differentiation of Neurons			
LSAMP	Male	0.68 (0.56, 0.84)	NA
	Female	1.19 (0.98, 1.45)	
NTM	Male	0.81 (0.71, 0.93)	NA
	Female	0.97 (0.91, 1.05)	
Lysosomal Homeostasis			
MBTPS1	Male	0.59 (0.38, 0.91)	NA
	Female	1.25 (1.03, 1.51)	
Prion Protein			
PRNP1	Male	1.23 (1.00, 1.51)	1.07 (0.85, 1.22)
	Female	1.04 (0.95, 1.14)	1.21 (0.89, 1.64)

## Data Availability

All relevant data are within the manuscript.

## Appendix A

**Table A1:** Primer Design for RT-qPCR.

Gene Family	Gene	Accession #	Forward Primer (5′→3′)	Reverse Primer (5′→3′)
Glucocorticoid Receptor	NR3C1	NM_012576.2	TGCTGGAGGTGATTGAACCC	TCACTTGACGCCCACCTAAC
Glutamate Signalling	GRIA2	NM_001083811.1	TACGGCATCGCCACACCTA	CTTGGAATCACCTCCCCCGC
	SLC1A2	NM_001035233.1	GTCAATGCCGCACACAACTC	GAATGGATGCAGGGGATGGT
Calcium Signalling	Ryr2	NM_001191043.1	CACTGAGAAGCCAAGACCGAA	CTCCATGACCCTCAGGGGA
	PLCH2	XM_017593885.1	AAATCCCAGAGTGCCCATCC	ATCGTTCCACCACAGGCAAA
	CACNB2	NM_053851.1	CAGCTGCACTGTCGGAATCT	AGTCATTCCATTTTTGCCCTGA
	CACNA1B	NM_001195199.1	GGGCTAATCTGCCCCAGAAG	GAGAGCCGCATAGACCTTCC
	CACNA1C	NM_012517.2	TCAAACGTCGCCACAGACAT	CGAAGGCCCGAATCATTGTG
	CAM2KA	NM_012920.1	AGACACCAAAGTGCGCAAAC	TTCCAGGGTCGCACATCTTC
Neural Transmission	SNAP25	NM_001270575.1	ATGTTGGATGAGCAAGGCGA	TCGGCCTCCTTCATGTCTTG
	Synaptophysin	NM_009305.2	CAGTTCCGGGTGGTCAAGG	ACTCTCCGTCTTGTTGGCAC
Growth and Differentiation	LSAMP	NM_017242.1	CACTGAGGAACACTACGGCA	ACCCGGGTCTGAAAAGGACT
of Neurons	NTM	NM_001357593.1	TCATGCTATTTGGCCCAGGT	GGAAGGGGCACTCACATCAA
Lysosomal Homeostasis	MBTPS1	NM_053569.1	GCGAGTAAACATCCCCCGAA	CCCAAATCTAGCAGGAGCCC
Prion Protein	PRNP1	NM_012631.3	CCAAGCCGACTATCAGCCAT	GCTTTTTGCAGAGGCCAACA
Reference Genes	CycA	NM_017101.1	CAGACGCCGCTGTCTCTTTTC	CGTGATGTCGAAGAACACGGT
	Ywhaz	NM_013011.3	GGCAGAGCGATACGATGACA	AAGATGACCTACGGGCTCCT
